# Are UMFA (un‐metabolized folic acid) and endocrine disruptor chemicals (EDCs) co‐responsible for sperm degradation? An epigenetic/methylation perspective

**DOI:** 10.1111/and.14400

**Published:** 2022-03-11

**Authors:** Yves Menezo, Patrice Clement, Kay Elder

**Affiliations:** ^1^ Laboratoire CLEMENT Paris France; ^2^ Bourn Hall Clinic Cambridge UK

**Keywords:** EDCs, epigenetic/methylation resetting, folic acid, sperm, UMFA

## CONFLICT OF INTEREST

The authors declare that there is no conflict of interest.

**FIGURE 1:**
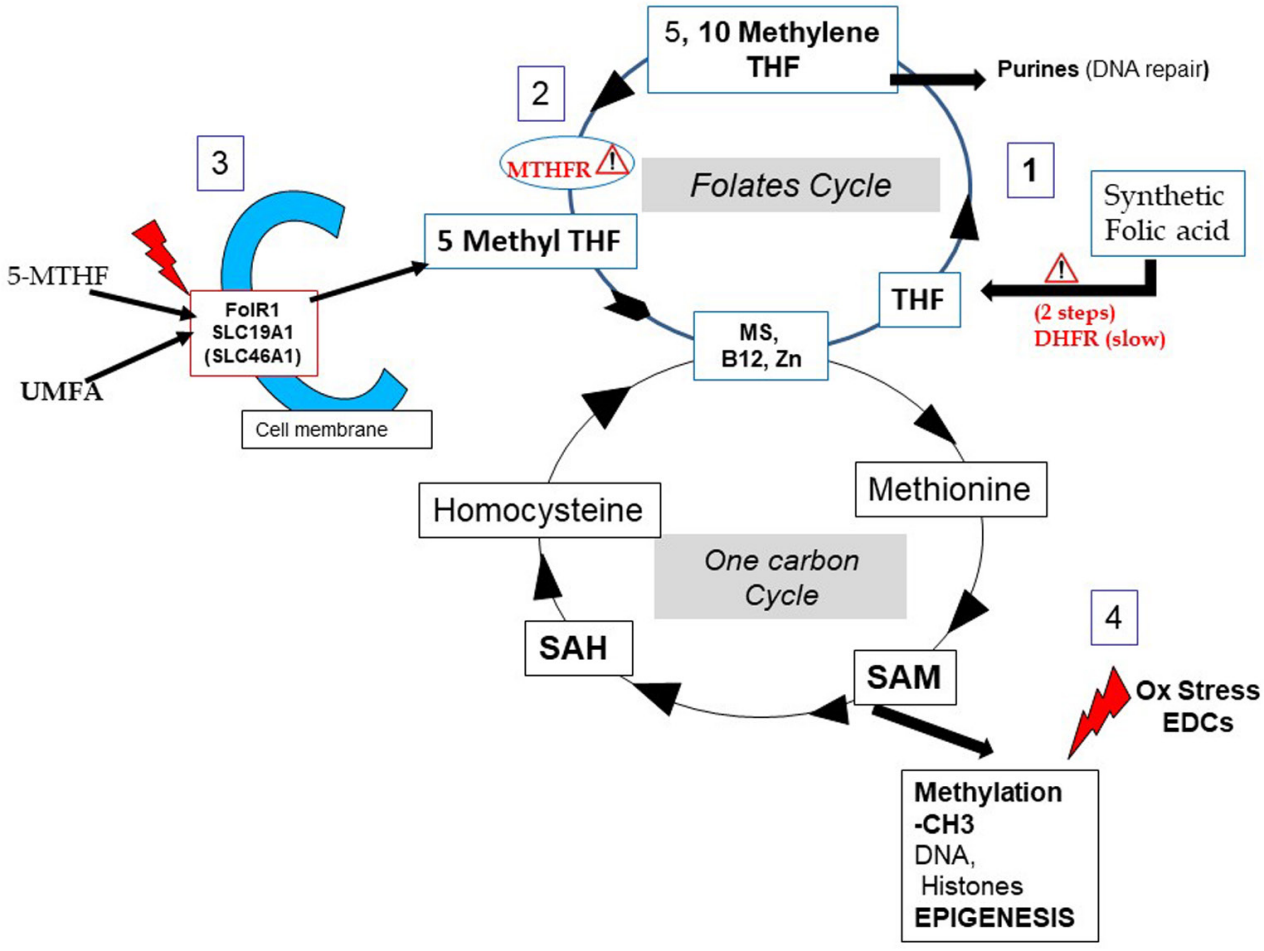
Perturbation in germline methylation/epigenetic process in the male embryo: the impact of UMFA and oxidative stress. (1) Weak DHFR activity (Bailey & Ayling, 2009) blocks the entry of UMFA into the folate cycle and leads to a Michaelis and Menten effect, a vicious cycle that further inhibits DHFR activity. (2) MTHFR SNPs (Methylenetetrahydrofolate Reductase single nucleotide polymorphisms), especially T677T, reduce MTHFR activity and impair the formation of 5‐MTHF (5 methyl tetrahydrofolate). The cycle pathway between THF and 5‐MTHF can be completely blocked; the cycle can be reversed with an accumulation of UMFA. (3) UMFA and active natural folate 5‐MTHF compete for receptor and transporters (Smith et al. 2017) in order to enter cells, and this exacerbates the situation by decreasing the availability of the natural folate for homocysteine regeneration. (4) EDCs (endocrine disruptor chemicals) exacerbate a process of undue demethylation via oxidative stress (Menezo et al., 2015). DHFR, dihydrofolate reductase; FolR1, folate receptor 1; MS, methionine synthase; MTHFR, methylenetetrahydrofolate reductase; SAH, S‐adenosyl homocysteine; SAM, S‐adenosyl‐Methionine; SLC19A1 and A1, folates transporters; THF, tetrahydrofolate

Dear Editor,

A decrease in sperm quality that is reflected in alteration of all classical sperm parameters is now observed in developed countries, and has been attributed to exposure to endocrine disruptor chemicals (EDCs). EDCs are also present in maternal body fluids and in embryos (Tang et al., 2020). They generate excessive amounts of reactive oxygen species and thereby cause oxidative stress, which exerts transgenerational effects linked to DNA methylation; this results in sperm epi‐mutations (Manikkam et al., 2013; Menezo et al., 2011; Tunc & Tremellen, 2009). Methylation reactions become suboptimal in terms of both ‘quality’ and ‘quantity’, with an effect on regulatory processes. In addition, supplementation with folic acid (FA) via nutritional fortification has been widely implemented in order to avoid nutritional deficiencies that cause neural tube defects (NTDs) during early pregnancy. This generalized increase in FA intake has led to the observation that un‐metabolized folic acid (UMFA) can now be found in umbilical cord blood, and in infants' circulation (Kalmbach et al., 2008; Plumptre et al., 2015; Sweeney et al., 2005). UMFA competes with 5‐methylene tetrahydrofolate (5‐MTHF) for folate receptor and transporter molecules; 5‐MTHF is the natural folate and is immediately available for recycling of homocysteine by the one carbon cycle (1‐CC) (Smith et al., 2017). This competition between natural folate and UMFA may have an impact on the resetting of methyl/epigenetic tags, which initially takes place very early in the male germline, and again during late embryogenesis (Blake et al., 2021; Ly et al., 2015). We discuss here the risk of abnormal germline epigenetic resetting imposed by the combined effects of EDCs and UMFA on the 1‐CC (Figure 1).

Epigenetic inheritance in the male germline occurs during embryogenesis; in females, epigenetic tags are reset during puberty. EDCs impair DNA methylation reactions, and this has resulted in a deterioration of gamete health. EDCs are found in foetal circulation in utero and in the placenta: they induce transgenerational inheritance of epigenetic perturbations that can lead to disturbances in growth, neurodevelopment, endocrinology and reproduction.

S‐adenosyl methionine (SAM), is the unique effector for methylation. Methylation of target molecules results in the formation of SAH (S‐adenosyl homocysteine) and release of homocysteine (Figure 1). Hcy is toxic to cells: it must be recycled to methionine via the one carbon cycle (1‐CC) supported by the folates cycle. Nutritional fortification programmes use FA supplementation to prevent vitamin B9 deficiencies. In order to fulfil a physiological function by entering the folates cycle, the synthetic FA molecule must first be reduced by DHFR to dihydrofolate (DHF) and then to tetrahydrofolate (THF) before it is converted to the biologically active 5‐MTHF. The inefficiency of these two enzymatic steps (Bailey & Ayling, 2009) leads to the accumulation of UMFA. The effect of this accumulation can be dramatic in patients carrying MTHFR SNPs, whose folate metabolism is further jeopardized by reduced efficiency of the MTHFR enzyme in producing 5‐MTHF. FA competes with all the folates, and especially with the immediately available natural folate 5‐MTHF, for folate receptor FolR1 and the folate transporter molecules. This means that a transitory shortage of active folate may occur during major essential steps of methylation/epigenetic resetting in the germline during embryogenesis (Blake et al., 2021; Ly et al., 2015). Recent studies (El Aarabi et al., 2015) provide confirmation: (1) folic acid severely disturbs the sperm methylome; and (2) this pathological feature is exacerbated in carriers of MTHFR SNPs (especially T677T). These observations should be considered in the context of embryo development.

The systemic UMFA concentration is directly influenced by folic acid intake; FA supplementation with >1000 µG during pregnancy may lower cognitive development in children (Valera‐Gran et al., 2017), often linked to epigenetic anomalies related to elevated homocysteine (Menezo et al., 2015). Nutritional FA fortification programmes have clearly decreased the incidence of neural tube defects, but public health policy should take into consideration the potential risks of transgenerational epigenetic disorders due to systemic UFMA, especially with the additional hazards due to EDCs in the environment of gametes and embryos. High doses of folic acid alter sperm methylome (El Aarabi et al., 2015; Ly et al., 2017), especially, but not only in MTHFR SNPs carriers. The type of folate (5‐MTHF vs FA) and the appropriate doses used in nutritional supplementation (avoiding high doses) should be re‐evaluated, especially in carriers of MTHFR SNPs, both before and during pregnancy.

## Data Availability

Research data are not shared.
